# Effect of Cyclotriphosphazene-Based Curing Agents on the Flame Resistance of Epoxy Resins

**DOI:** 10.3390/polym13010008

**Published:** 2020-12-22

**Authors:** Lucie Zarybnicka, Jana Machotova, Radka Kopecka, Radek Sevcik, Martina Hudakova, Jaroslav Pokorny, Jiri Sal

**Affiliations:** 1 Institute of Theoretical and Applied Mechanics of the Czech Academy of Sciences, Centre Telc, Prosecka 809/76, 190 00 Prague, Czech Republic; sevcik@itam.cas.cz; 2 Institute of Chemistry and Technology of Macromolecular Materials, Faculty of Chemical Technology, University of Pardubice, Studentska 573, 532 10 Pardubice, Czech Republic; jana.machotova@upce.cz; 3 Department of Chemistry, Faculty of Science, Masaryk University, Kotlarska 267/2, 611 37 Brno, Czech Republic; radkabacovska@centrum.cz; 4 The Institute of Technology and Business in Ceske Budejovice, Okruzni 517/10, 370 01 Ceske Budejovice, Czech Republic; jaroslav.pokorny@mail.vstecb.cz (J.P.); sal@mail.vstecb.cz (J.S.); 5 Fire Research Institute, Ministry of Interior of the Slovak Republic, Roznavska 11, 83 104 Bratislava, Slovakia; Martina.Hudakova@minv.sk

**Keywords:** cyclotriphosphazene derivative, synthesis, flame resistance, curing agent, epoxy resin, thermal degradation, cone calorimeter

## Abstract

Epoxy resins are characterized by excellent properties such as chemical resistance, shape stability, hardness and heat resistance, but they present low flame resistance. In this work, the synthesized derivatives, namely hexacyclohexylamino-cyclotriphosphazene (HCACTP) and novel diaminotetracyclohexylamino-cyclotriphosphazene (DTCATP), were applied as curing agents for halogen-free flame retarding epoxy materials. The thermal properties and combustion behavior of the cured epoxy resins were investigated. The obtained results revealed that the application of both derivatives significantly increased flame resistance. The epoxy resins cured with HCACTP and DTCATP exhibited lower total heat release together with lower total smoke production compared to the epoxy materials based on conventional curing agents (dipropylenetriamine and ethylenediamine). Comparing both derivatives, the HCACTP-cured epoxy resin was found to provide a higher flame resistance. The designed novel class of epoxy materials may be used for the preparation of materials with improved flame resistance properties in terms of flame spreading and smoke inhibition.

## 1. Introduction

In the modern polymer industry, epoxy resins have been extensively used as advanced composite matrices in a wide field of applications due to their attractive properties such as high adhesion, excellent mechanical properties, good chemical and corrosion resistance, damage tolerance, impact resistance, outstanding dimensional stability and remarkable electric insulating properties [1,2,3,4,5,6]. Nonetheless, the low flame resistance of conventional epoxy resins [7] limits their application in electronic and microelectronic industries where a high flame resistance and a remarkable self-extinguish ability are required. The traditional bromine-containing compounds that have been widely utilized to prepare flame retarding materials are currently not preferred for their health and safety hazards [8,9,10,11]. Therefore, an extensive study addressing this problem has been focused on the achieving a high flame resistance of epoxy resins by using nonhalogenated flame retardants [12]. Among them, phosphazene-based compounds bearing simultaneously phosphorus and nitrogen atoms in their structure have attracted a great attention [13,14,15,16,17,18,19,20,21,22]. This class of products brings a synergistic flame retarding effect: phosphorus atoms act as radical scavengers and quenchers [23] and nitrogen atoms release inert gaseous by-products that create a porous network providing thermal insulation during the combustion [24,25,26]. Other flame retardants include nanocomposites [27], silicon-based compounds [28] and metal-based flame retardants [29,30,31]. Recently, considerable efforts have been focused on the development of halogen-free flame retardants for epoxy resins [32,33,34,35] such as a spiro-phosphorus (P)-based compound [36] or phosphaphenanthrene/benzimidazole-based agent [37]. Another phosphorus-based flame retardant was melamine phenyl phosphate [38] which greatly enhanced the flame retardancy and suppressed smoke evolution during burning of cured epoxy resin.

Hexachlorocyclotriphosphazene is an adaptable starting compound for the synthesis of a variety of phosphazene-based derivatives because of presence of two chlorine atoms attached [39,40,41,42,43]. Thus, different additives [44,45,46,47] and reactive types [18,48,49,50,51,52,53] of compounds with varied substituent groups could be designed and synthesized. If the cyclotriphosphazene-based derivatives have been incorporated into the polymer network, a significant flame retardancy and self-extinguishability owing to the unique molecular structure of cyclotriphosphazene based on alteration of phosphorous and nitrogen atoms in a conjugative mode were observed [54,55,56,57,58]. However, only a few investigations [59,60] have been reported on the application of cyclotriphosphazene derivatives in the combined role of a flame retardant and curing agent for epoxy resins.

In this work, two cyclotriphosphazene-based derivatives, namely hexacyclohexylamino-cyclotriphosphazene (HCACTP) [61,62] and novel diaminotetracyclohexylamino-cyclotriphosphazene (DTCATP) have been successfully synthetized. These derivatives offer several advantages: halogen-free flame retarding epoxy materials can be produced with better chemical stability and increased flame resistance by introducing the flame retardant in the curing process. The derivative was prepared in toluene solution in the presence of trimethylamine in the work of Richards et al. In our work, an excess of cyclohexylamine instead of triethylamine was used and, moreover, tetrahydrofuran was used as solvent. The chemical structure of the cyclotriphosphazene derivatives was characterized by ^31^P nuclear magnetic resonance (NMR), elemental analysis (EA), mass spectroscopy (MS) and Fourier transform infrared spectroscopy (FTIR). Then, a curing process of a conventional epoxy resin based on diglycidyl ether of bisphenol A (DGEBA) with the synthetized HCACTP and DTCATP, was monitored using differential scanning calorimetry (DSC). The selected samples were observed under scanning electron microscope (SEM). Finally, thermal properties and combustion behavior of epoxy materials cured with HCACTP, DTCATP and two conventional aliphatic amine curing agents, ethylenediamine (EDA) and dipropylenetriamine (DPTA), were evaluated by means of DSC, thermogravimetry analysis (TGA) and cone calorimeter measurements.

## 2. Materials and Methods

### 2.1. Materials

Cyclohexylamine (Sigma-Aldrich, Schnelldorf, Germany, purity 99.0%), tetrahydrofuran (THF, Penta Chemicals, Chrudim, Czech Republic, purity 99.8%), hexachlorocyclotriphosphazene (HCCTP, Sigma-Aldrich, Schnelldorf, Germany, purity 99.0%) were used for the synthesis of phosphazene derivatives. THF was stored under anhydrous conditions using activated molecular sieves. As epoxy resin, diglycidyl ether of bisphenol A (DGEBA) (CHS-EPOXY 520, Spolchemie, Usti nad Labem, Czech Republic) with an epoxy equivalent weight (EEW) of about 190 g/equiv. [63,64,65,66] and E-index 5.27 mol/kg was selected. Ethylenediamine (EDA) (purity 99.5%) and dipropylenetriamine (DPTA) (purity 98.0%) were obtained from Sigma-Aldrich, Schnelldorf, Germany.

### 2.2. Synthesis of HCACTP

**Figure 1:**
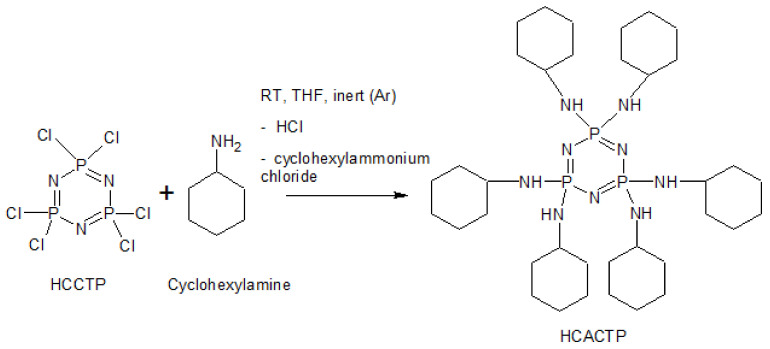
Scheme of the reaction pathway of the HCACTP synthesis.

All steps of the synthesis were performed under anhydrous conditions in an inert argon atmosphere using Schlenk containers. HCCTP (0.0086 mol, 2.989 g) was dissolved in THF (50 mL). Cyclohexylamine (0.086 mol, 8520 g) was dissolved in 50 mL of THF and then added dropwise to the HCCTP solution under continuous stirring. The mixture was further stirred at room temperature for three days. After that, the formed salt was filtered, three times washed with THF and the final product was precipitated in distilled water. After the filtration, HCACTP was vacuum-dried at 30 °C for 24 h. Finally, light white product (yield: 89.5%, purity: 99.5%) was prepared (seen in Figure 1). Richards et al. [61] already described the procedure of the synthesis of HCACTP derivate that proceeded in toluene solution in the presence of trimethylamine. The yield of the prepared HCACTP according to [61] was only 72%. To the contrary, in this work, an excess of cyclohexylamine instead of triethylamine was used and, moreover, tetrahydrofuran was used as the solvent. The final white solid product was characterized by ^31^P NMR, EA, MS, FTIR and TGA.

### 2.3. Synthesis of DTCATP

**Figure 2:**
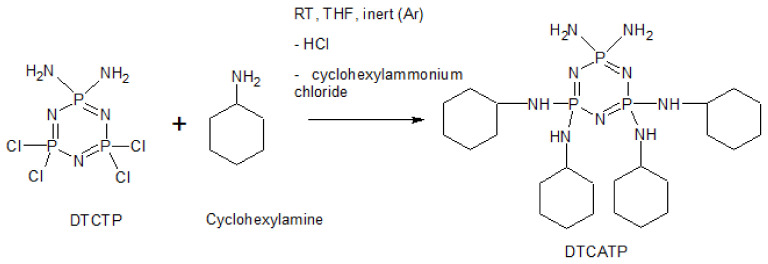
Scheme of the reaction pathway used for the synthesis of DTCATP.

The whole synthesis was performed under anhydrous conditions in an inert argon atmosphere using Schlenk containers. Diaminotetrachloro-cyclotriphosphazene (DTCTP) as the starting compound was prepared according to the procedure well described in the literature in an ammonia solution of dried diethyl ether [67]. The yield was 72.6 wt % and the targeted DTCTP was characterized by a melting point of 162.5 °C and ^31^P NMR. DTCTP (0.008 mol, 4.460 g) was dissolved in 30 mL of THF. Cyclohexylamine (0.097 mol, 9.619 g) was dissolved in THF (50 mL) and then added dropwise to DTCTP solution under continuous stirring. The mixture was further stirred at room temperature for 3 days. Then the salt was filtered, three times washed with THF and the final product (yield: 81.2%, purity: 99.0%) was precipitated in distilled water. After the filtration, DTCATP was vacuum-dried at 30 °C for 24 h. The reaction scheme is shown in Figure 2. The final pale-yellow solid product was characterized by ^31^P NMR, EA, MS, FTIR and TGA.

### 2.4. Preparation of Epoxy Materials

DGEBA was cured using HCACTP and DTCATP, respectively. EDA and DPTA were used as the references. The epoxy resin was mixed with a particular curing agent in the respective amount calculated according to the Equation (1):

g of curing agent/kg of DGEBA = E-index × *H_ekv_* × 1.1 (1)

where E-index of DGEBA is 5.27 mol/kg and *H_ekv_* (g/mol) is the hydrogen equivalent of the particular curing agent. *H_ekv_* values for the utilized curing agents are shown in Table 1. The value of 1.1 corresponds to 10% excess of the curing agent.

The mixture was stirred constantly to become homogeneous and then was centrifuged at 3000 rpm for 3 min to remove trapped air. The curing process was carried out in a silicone mold to obtain the thermosetting epoxy resin. The precuring was performed according to curing conditions listed in Table 1. The precuring conditions for HCACTP and DTCATP were determined according to the DSC tracings of the respective curing system, whereas the precuring conditions for both control amine-based curing agents were set the same such as for the less reactive cyclotriphosphazene-based curing agent. The postcuring was carried out at 120 °C for 3 h. At the end of the curing procedure, the cured system was cooled gradually to ambient temperature to avoid stress cracking.

### 2.5. Measurements and Characterization

^31^P nuclear magnetic resonance (NMR) spectra were measured by a Bruker Avance DRX 300 instrument (Billerica, MA, USA) at the frequency of ^31^P 202.46 MHz with 85% H_3_PO_4_ as the external standard. The samples were sealed in Simax tubes (diameter 4 mm) and inserted in NMR cuvettes (diameter 5 mm) filled with D_2_O (external lock). The spectra were measured in the coaxial NMR cuvette system.

EA was performed using a FLASH Organic 2000 Elemental Analyzer (Thermo Fisher Scientific, Waltham, MA, USA). Chlorine was determined by burning using Schöniger method [68]. The reliability of this method was tested using *o*-chlorobenzoic acid as the standard.

Melting points of newly synthesized derivatives were determined using a Kofler bench (Nagema, Berlin, Germany). The heating rate was 5 °C/min (range 50–260 °C).

The amine number of the curing agent (mg KOH/g) was determined by potentiometric titration according to EN 1887-2 and the theoretical amine number was calculated according to the Equation (2):

Amine number _theor._ = [(*X* × 56.11)/*M*] × 1000 (2)

where *X* is the number of amine groups in the molecule and *M* is the molecular weight of the compound.

MS was performed using Agilent Technologies instrument (MSD Model 5975B) with a probe for direct entry and a mass selective detector operating in the electron impact ionization mode with the ionization energy of 70 eV. The samples were analyzed as 0.01 wt % solutions in methanol.

FTIR spectra were recorded using attenuated total reflection (ATR) module of a Nicolet IS50 infrared spectrometer in the spectral range from 500 to 3500 cm^−1^ with 0.09 cm^−1^ resolution.

DSC was performed according to ISO 11357-2 using a Perkin-Elmer DSC (Perkin-Elmer, Waltham, MA, USA). The glass transition temperature (*T_g_*) was measured in a nitrogen atmosphere at a heating rate of 20 °C/min. Samples weights were in the range from 2 to 5 mg. For monitoring the curing process, DGEBA were mixed properly (ratios are reported in Table 1) with HCACTP or DTCATP. The mixture was immediately placed in the DSC device, followed by heating the sample from 40 °C to 130 °C at a heating rate of 10 °C/min.

The conversion of DGEBA precuring with the phosphazene derivatives was determined using DSC tracings according to the procedure described in [60]. A mixture of DGEBA and a particular curing agent was stirred to obtain homogenized mixture that was used for precuring for a given time interval at a selected optimal curing temperature (100 °C for HCACTP and 70 °C for DTCATP). After that, the mixture was placed in the DSC device, followed by heating the sample from 40 to 130 °C at a heating rate of 10 °C/min. The curve integral was used to calculate the curing heat. The precuring conversion *α* was calculated from the Equation (3):

*α* = 100 × (|*H*_0_| − |*H_t_*|)/|*H*_0_| (3)

where *H*_0_ is the curing enthalpy of the sample without precuring and *H_t_* is the enthalpy of the sample precured for a given time period at a selected temperature.

TGA was carried out with a Sartorius balance BP210E S using a quartz ampoule at a heating rate of 5 °C/min from 25 to 950 °C under air atmosphere to keep the same conditions as for Cone calorimeter testing described below. Decomposition steps determined in TGA curves were characterized by weight loss values and temperatures at rapid weight loss, the latter being assigned to the inflexion point (midpoint) of the particular step change. The values of molecular weight loss for each degradation step were calculated considering the theoretical molecular weight of the respective cyclotriphosphazene derivative.

The cryo-fractures of epoxy materials were compared by means of a high-resolution SEM Quanta 450 FEG (FEI) using a secondary electron detector. Analyses were performed at the accelerating voltage between 5 and 20 kV. Samples were placed on carbon tape and gold coated with a 7 nm thick layer.

The crosslinking density of epoxy materials was evaluated from swelling experiments performed on dry gel polymer samples (around 0.2 g) which were immersed in toluene at 25 °C for one week. A swelling time of one week was chosen on the basis of the test results on several samples which showed no significant changes after one week of immersion in toluene. At the end of the immersion period, the sample was removed, rapidly blotted with tissue and transferred to the weighting bottle to obtain the swollen weight of the sample. The theory of Flory and Rehner [69] was used to calculate the average molecular weight between crosslinks (*M_c_*) and the crosslinking density (expressed as moles of crosslinks per cm^3^ of polymer network), as given in the following equations:

(4) $$M_{c}=\;\frac{V_{1}\rho_{p}\left[\phi^{1/3}-\;\phi /2\right]}{-\left[ln\left(1-\phi \right)+\;\phi +\;\chi \phi^{2}\right]}$$

(5) $$\phi =\;\frac{W_{p}\rho_{s}}{W_{p}\rho_{s}+W_{s}\rho_{p}}$$

(6) $$\chi =0.34+\;\frac{V_{1}}{\mathrm{RT}}{\left(\delta_{1}-\;\delta_{2}\right)}^{2}$$

Crosslinking density = *ρ*_p_/*M_c_* (7)

where: *V*_1_ is the molar volume of toluene (106.3 cm^3^/mol); *ρ*_p_ is the density of polymer that was calculated to be 1.165 g/cm^3^ for the DGEBA; *φ* is the volume fraction of the gel polymer in the swollen gel; W_p_ and W_s_ are the weight fractions of the gel polymer and toluene in the swollen gel, respectively; *ρ*_s_ is the density of solvent (0.8669 g/cm^3^); *χ* is the polymer and solvent interaction parameter; *δ*_1_ is the solubility parameter of polymer that was calculated to be 11.05 [cal/cm^3^]^1/2^ for DGEBA resin [70,71]; and *δ*_2_ is the solubility parameter of toluene, 8.9 [cal/cm^3^]^1/2^; R is gas constant (8.314 J/(K⋅mol)); T is temperature (K).

Cone calorimeter measurements were performed using Fire Testing Technology cone calorimeter (West Sussex, UK) according to the ISO 5660-1 standard under an external heat flux of 50 kW/m^2^. The dimension of samples were 100 mm × 100 mm × 5 mm. The average values of heat release rate, effective heat of combustion, CO yield and CO_2_ yield were obtained during the time period from 0 to 489 s. Three replicates were measured for each testing specimen and the reproducibility of error values of were found to be within ±5%.

## 3. Results and Discussion

### 3.1. Characterization of Cyclotriphosphazene Derivatives

The synthetic schemes of the cyclotriphospazene-based derivatives are illustrated in Figure 1 and Figure 2. Results of EA confirmed full substitution of chlorine groups with cyclohexylamine functional groups and full substitution of amine and cyclohexylamine functional groups for HCACTP and DTCATP, respectively (Table 2). No chlorine was detected in both samples. The determined concentrations of C, H, N and P were found to be in a good correlation with theoretical values as represented in Table 2. Similarly, no significant differences were found between the experimental and theoretical values of amine number (see Table 3), which corresponds to high purity of the synthetized cyclotriphosphazene derivatives.

**Figure 3:**
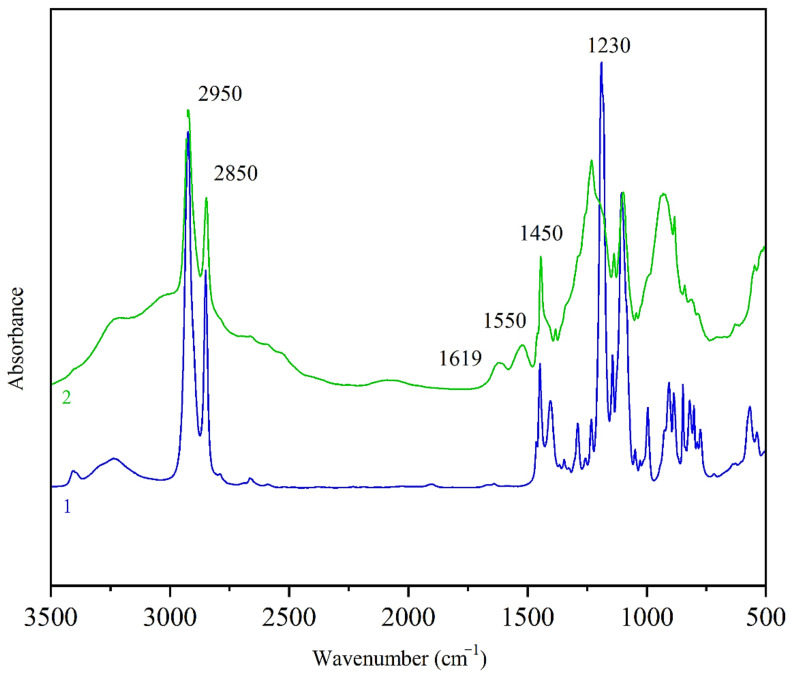
FTIR spectra of HCACTP (spectrum 1) and DTCATP (spectrum 2).

The measured FTIR spectra of the synthetized derivatives are depicted in Figure 3. The presence of the absorption bands around 900–1000 cm^−1^, corresponding to the P=N stretching vibration of the phosphazene groups [72], is clearly visible. Furthermore, the absorption bands at 3200 and 3400 cm^−^^1^ corresponding to the absorption of N–H bonds [72] and the bands at 2950 and 2850 cm^−1^ corresponding to the presence of C–H bonds were detected [72]. The FTIR spectrum of the second reagent, cyclohexylamine, was already described in literature [73] and comprises the absorption bands at around 3360, 3340 and 1619 cm^−1^ that are ascribed to the presence of N–H bonds [73]. These bands appear as doublets in the spectrum reflecting the symmetric and asymmetric stretching of primary amines molecules. On the contrary, secondary amines (–N–H–) show only one singlet at around 1550 cm^−1^ [74]. Due to a successful conversion of reagents into the final HCACTP derivative, no bands of the N–H bond are visible in the recorded FTIR spectrum (see Figure 3, spectrum 1). On the other hand, in the FTIR spectrum of DTCATP, the absorption band at around 1619 cm^−1^ was detected due to the presence of 2 primary amines groups in the DTCTP structure that stayed preserved after the reaction with cyclohexylamine, as illustrated in the reaction scheme depicted in Figure 2.

Stretching vibrations of P–N–C bonds were observed at wavenumbers in the range of 1115–1186 cm^−1^ for HCACTP and in the range 1100–1115 cm^−1^ for DTCATP. Deformation vibrations bands of CH_x_ groups (x = 1–3) were detected in the region around 1400–1450 cm^−1^ in the case of HCACTP, and in the region of 1380–1450 cm^−1^ in the case of DTCATP. Stretching vibrations of ‘endocyclic’ P–N bonds were presented in the range of 1230–1289 cm^−1^ for DTCATP and at around 1230 cm^−1^ for HCACTP. The absence of P-Cl bonds was confirmed because no absorption bands around 600 cm^−1^ were detected.

**Figure 4:**
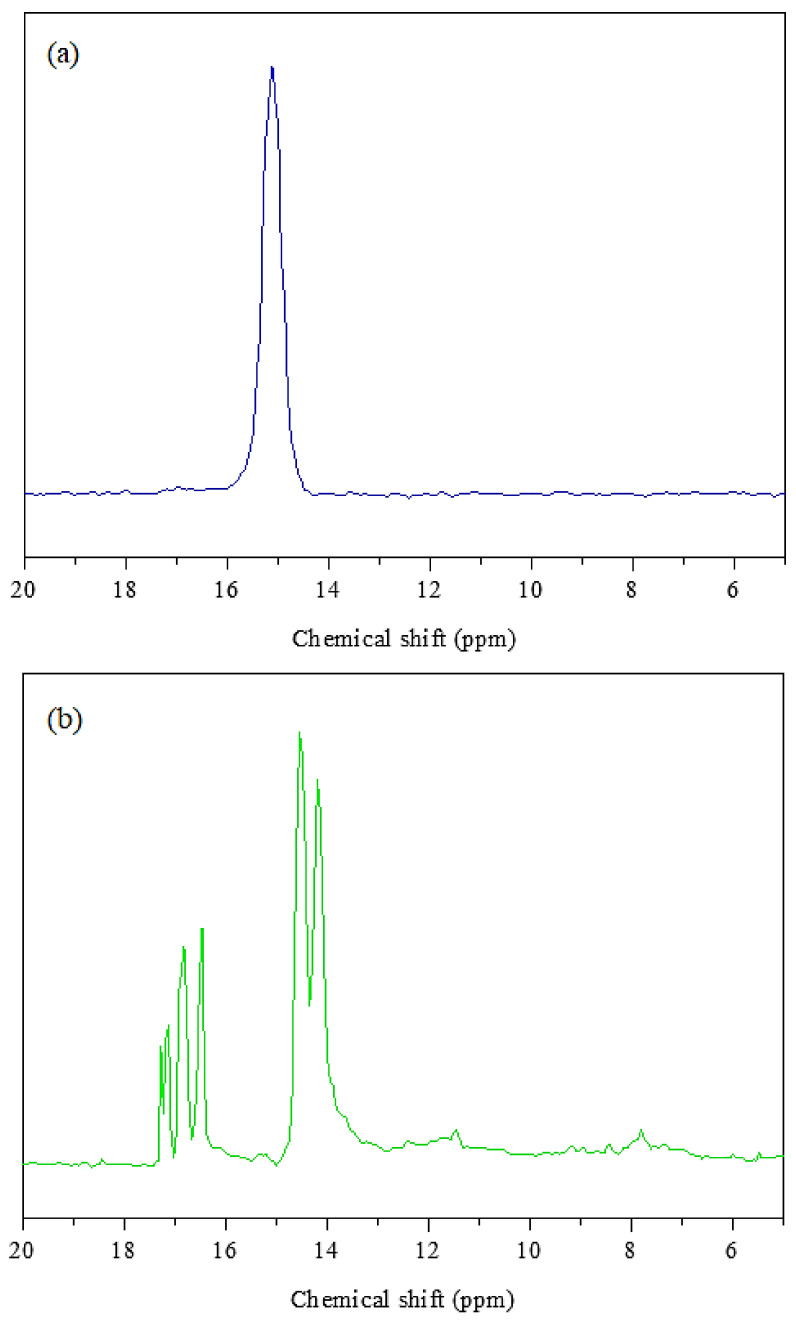
^31^P NMR spectra of HCACTP (**a**) and DTCATP (**b**).

NMR spectra of final derivates are shown in Figure 4. HCACTP contains chemically equivalent phosphorus atoms in its structure, it corresponds to only one singlet with δ = 15.13 ppm in the spectrum (Figure 4a). In the case of DTCATP derivative (Figure 4b), a doublet at 14.38–14.39 ppm corresponding to two amine groups on cyclotriphosphazene unit and a triplet at 17.03–17.09 ppm corresponding to four cyclohexylamine groups were found with an intensity of 1:2. The ^31^P NMR spectra of HCCTP and DTCTP reagents were already reported [74]. One singlet with δ = 20.12 ppm and two multiplets (doublet for –P–Cl_2_ with chemical shift δ (D) = 18.3 ppm and triplet of –P–(NH_2_)_2_ with δ (T) = 9.03 ppm) were found for HCCTP and DTCTP reagents, respectively. Due to absence of these signals in measured NMR spectra of HCACTP and DTCATP, full substitution of chlorine groups in molecular skeletons was confirmed.

Molecular weights of HCACTP and DTCATP, determined using MS, were found to be in a good correlation with theoretical values. Molecular weights of HCACTP and DTCATP were detected to be 724.00 g/mol (theoretical value 723.94 g/mol) and 560.0 g/mol (theoretical value 559.65 g/mol), respectively.

**Figure 5:**
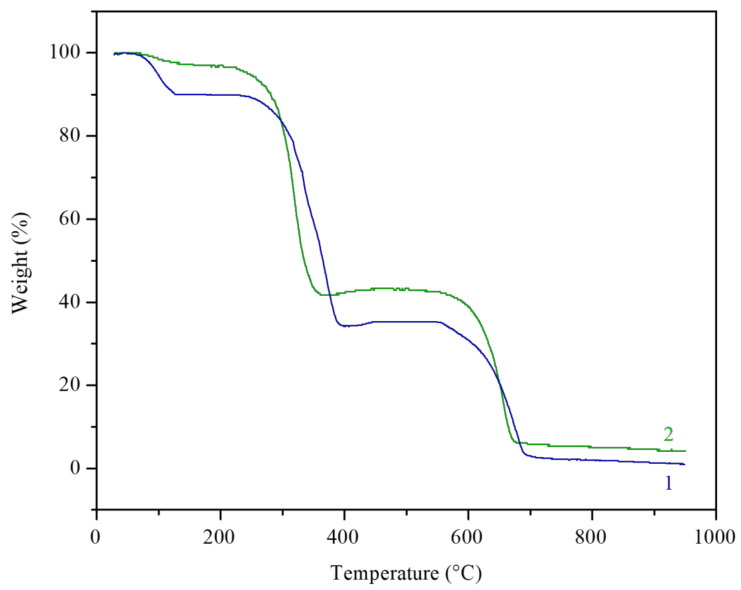
Thermogravimetry (TG) thermograms of HCACTP (curve 1) and DTCATP (curve 2).

**Figure 6:**
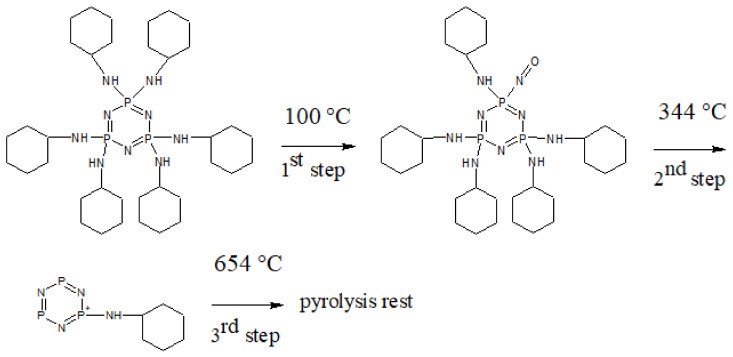
The thermo-oxidative degradation scheme for HCACTP.

**Figure 7:**
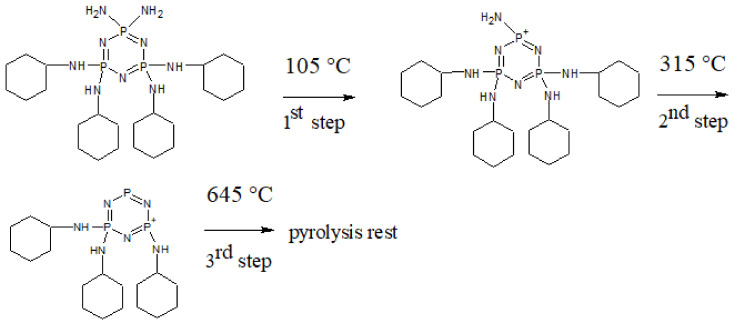
The thermo-oxidative degradation scheme of DTCATP.

The effect of molecular structure of the prepared cyclotriphosphazene derivatives on their thermal stability was evaluated by TGA under air atmosphere. Figure 5 depicts the TG thermograms for both cyclotriphosphazene derivatives. The results are summarized in Table 4 and Table 5. Both derivatives were exhibited a three-step thermal degradation. Based on the knowledge of dissociation energies of particular bonds [75] present in HCACTP and DTCATP, it can be assumed that during the thermo-oxidative decomposition, the cyclohexylamine groups were cyclically decomposed into cyclohexane and ammonia together with a subsequent decomposition of the cyclotriphosphazene unit (Figure 6 and Figure 7). Comparing the results of molecular weight loss with the molecular weight of the considered decomposition products (17.03 and 84.16 g/mol for ammonia and cyclohexane, respectively) and with the molecular weight of the particular functional groups (16.03 and 98.17 g/mol for amine and cyclohexylamine group, respectively), it can be assumed that the thermo-oxidative decomposition of HCACTP proceeded as follows. In the first step, cyclohexane was apparently released from one cyclohexylamine group followed by oxygen attachment to the nitrogen residue of the cyclohexylamine group. After that, the elimination of four cyclohexylamine groups probably proceeded in the same manner in the second step. Finally, the remaining cyclohexylamine group and cyclotriphosphazene skeleton were decomposed in the third step. In the case of DTCATP thermo-oxidative degradation, the decomposition process seemed to proceed as follows. In the first step, one amine group was removed producing ammonia. In the second step, the additional amine group was eliminated and three cyclohexylamine groups were probably decomposed resulting in cyclohexane release and oxidation of nitrogen residuals. Finally, the remaining one cyclohexylamine group and the phosphazene base were decomposed in the third step. When comparing the thermo-oxidative stability of both synthesized cyclotriphosphazene derivatives (see Table 5), it was found that DTCATP was more stable than HCACTP, which correlates well with dissociation energies of the particular bonds forming the molecular structures of the derivatives.

### 3.2. Monitoring of Curing Process

In general, the curing of epoxy resin by amine curing agents involves two major addition reactions; the active hydrogen in primary amine reacts with an epoxy group to form secondary amine and the secondary amine reacts with another epoxy group to cure [76]. Epoxy resins with two terminal epoxide groups can be cured with agents such as diamines, anhydrides or isocyanates [77]. The amine curing agent must have more than three active hydrogen atoms and two amino groups in a molecule so that the cured epoxy resin becomes cross-linked polymer. The same principles are also valid for the curing using HCACTP and DTCATP derivatives comprising primary and secondary amine groups in their structures. Reference epoxy materials cured with EDA and DPTA followed the curing procedures already described elsewhere [78,79].

**Figure 8:**
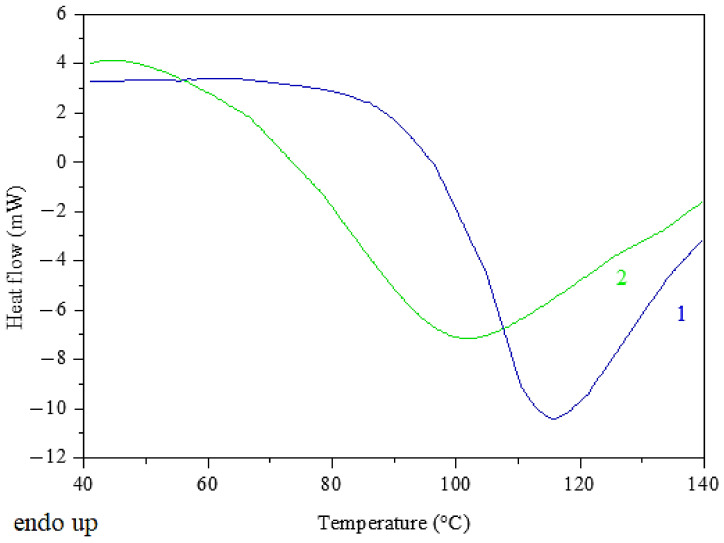
Differential scanning calorimetry (DSC) records of start of curing DGEBA with HCACTP (curve 1) and DTCATP (curve 2).

The curing process of DGEBA with HCACTP and DTCATP was investigated using DSC tracings (Figure 8). The results revealed that DTCATP was a more reactive curing agent than HCACTP. The significant curing reaction (demonstrated by heat evolution) of DTCATP was detected at temperatures above 50 °C. However, the curing reaction of HCACTP derivative was measured at the temperatures above 80 °C. These experimental findings are in a good agreement with the theoretical assumption based on the fact that the primary amine groups are more reactive than the secondary groups (DTCATP contains two primary amine groups and four secondary amine groups in the molecule, whereas HCACTP contains six secondary amine groups). To ensure a sufficient rate of the DGEBA curing reaction, the optimal precuring temperature for further experiments was set to 70 and 100 °C for DTCATP and HCACTP, respectively.

**Figure 9:**
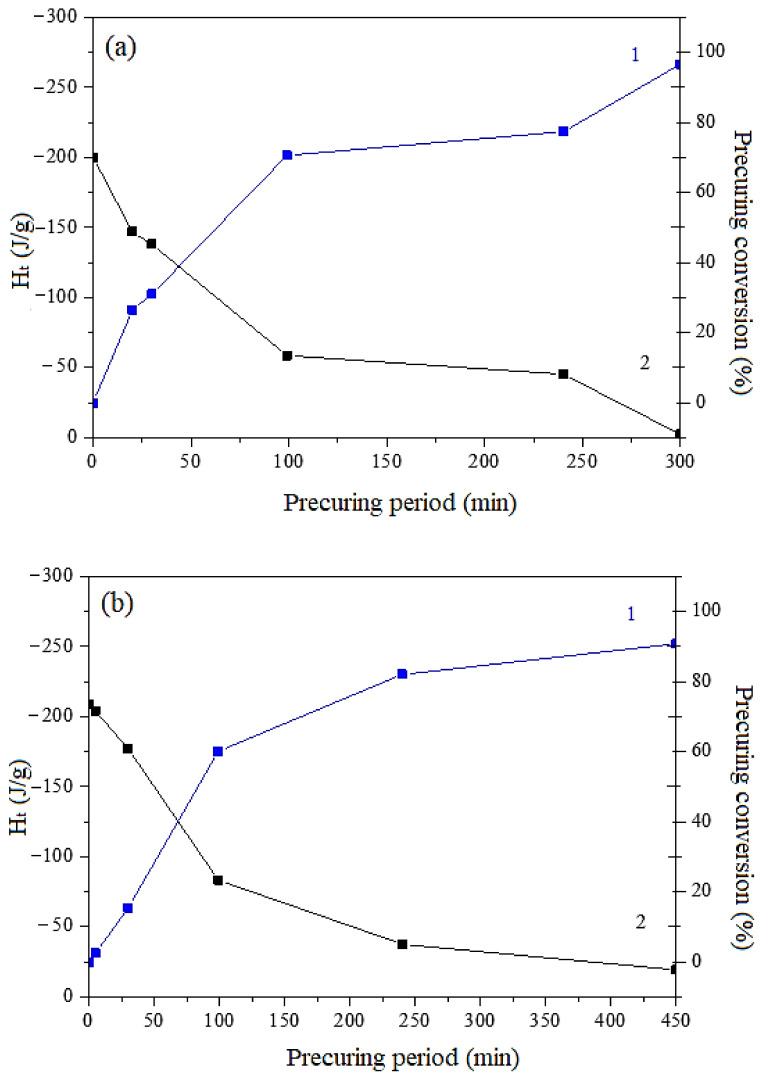
Curing heat (curves 1) and conversion (curves 2) of reactions of DGEBA with HCACTP (**a**) and DTCATP (**b**).

Furthermore, the conversion of DGEBA precuring process with HCACTP and DTCATP derivatives, at the respective optimal temperature was determined based on the extent of curing heat released during DSC measurements. The results are listed in Figure 9. It visible that for both phosphazene-based curing agents a sufficiently high (around 80%) conversion of the curing reaction was achieved after 4 h of curing time at the selected temperature. Thus, the time interval of 4 h was chosen as the optimal duration of the precuring process for both types of the cyclotriphosphazene derivatives.

### 3.3. Characterization of Epoxy Materials

DGEBA-based epoxy resin cured with HCACTP, DTCATP and with two conventional aliphatic amine curing agents (EDA and DPTA) as a reference samples were evaluated and compared from the point of view of thermal stability and combustion behavior properties. A detail description of two-step curing process of DGEBA with the curing agents is reported in Table 1. The postcuring process was same for all investigated samples—3 h at 120 °C.

**Figure 10:**
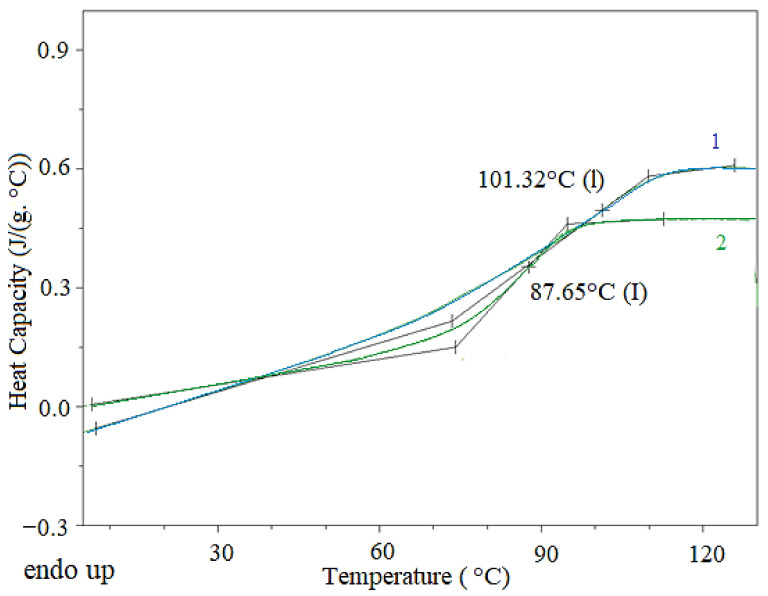
DSC records for DGEBA cured with both derivatives, HCACTP (curve 1) and DTCATP (curve 2).

The curing efficiency of the prepared cyclotriphosphazene derivatives was evaluated on the basis of glass transition temperature (*T_g_*) and the change of crosslinking density (see Table 6, Figure 10). According to the literature, DGEBA-based thermosets cured with linear amines usually exhibit *T_g_* values close to 110 °C [80]. The resulting DGEBA-based epoxy materials cured with HCACTP, DTCATP possessed a significantly reduced *T_g_*. In accordance with the expectations, the curing efficiency of a respective curing agent HCACTP and DTCATP were influenced by its molecular bulkiness, which governs the mobility, and by the number of primary and secondary amine groups per molecule. Therefore, the control amine curing agents (EDA and DPTA) provided more densely crosslinked epoxy materials with elevated *T_g_* in contrast to both phosphazene curing agents, where EDA was shown to be the most effective while HCACTP the least effective. When comparing both phosphazene curing agents, DTCATP was found to be a more effective curing agent, probably due to the presence of primary amine groups providing a higher reactivity and crosslinking density. The properties of uncured DGEBA-based thermosets may be found elsewhere [60].

**Figure 11:**
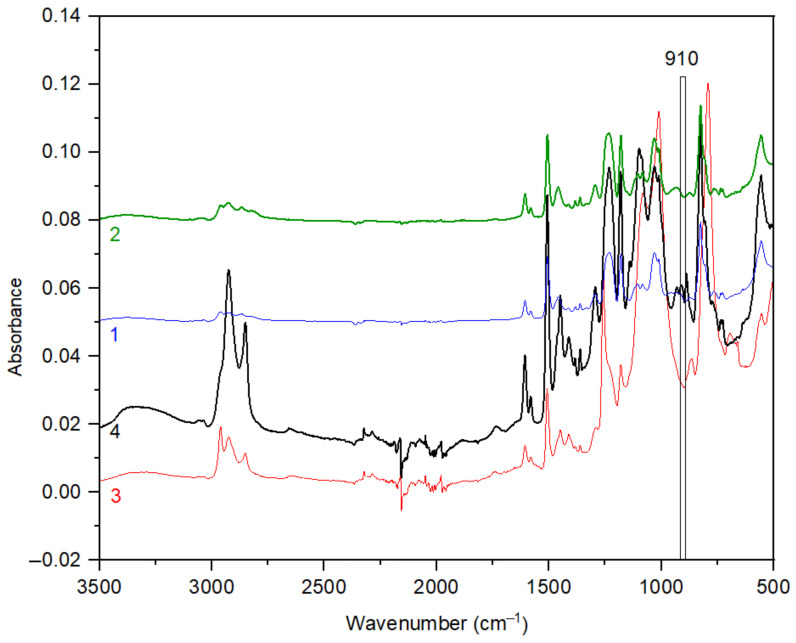
FTIR spectra of DGEBA cured with HCACTP (curve 1), DTCATP (curve 2), EDA (curve 3) and DPTA (curve 4).

FTIR spectra of samples after curing reaction are shown in Figure 11. The intensity of the absorption band at 910 cm^−1^ (corresponding to the stretching vibration of C–O bonds present in glycidyl groups [81]) was found to be negligible for all samples. Thus, it can be stated that epoxy groups in DGEBA were fully consumed during reaction with amine-based curing agents.

**Figure 12:**
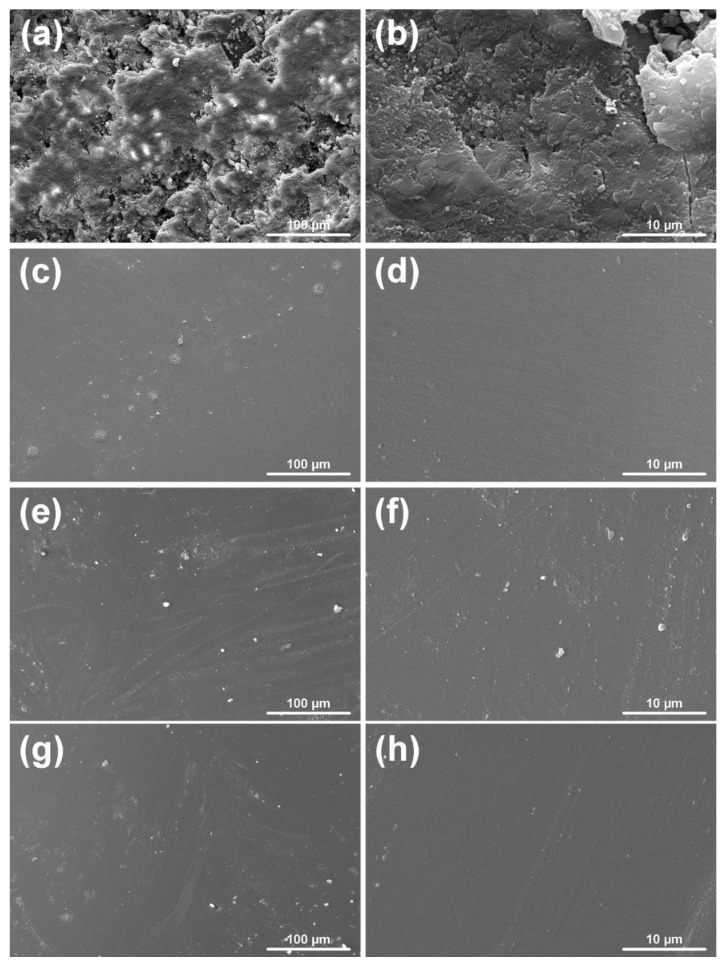
SEM images of the epoxy resin cured with: HCACTP (**a**,**b**); DTCATP (**c**,**d**), EDA (**e**,**f**); DPTA (**g**,**h**). Images observed at 1000× magnification (on the left) and 10,000× magnification (on the right) are depicted.

Selected samples after curing reaction were observed under SEM to verify the surface compactness (Figure 12). A compact surface was observed in the case of densely crosslinked samples cured using EDA, DPTA and DTCATP, while the HCACTP-cured epoxy resin exhibited a structure with many micropores as visible in Figure 12a,b. The reason of high roughness of HCACTP samples could be ascribed to the presence of six cyclohexyl groups bounded to the nitrogen atoms in the skeleton of this compound. Such complex structure with high steric hindrance might probably affect the proper preparation of the cured epoxy resin.

**Figure 13:**
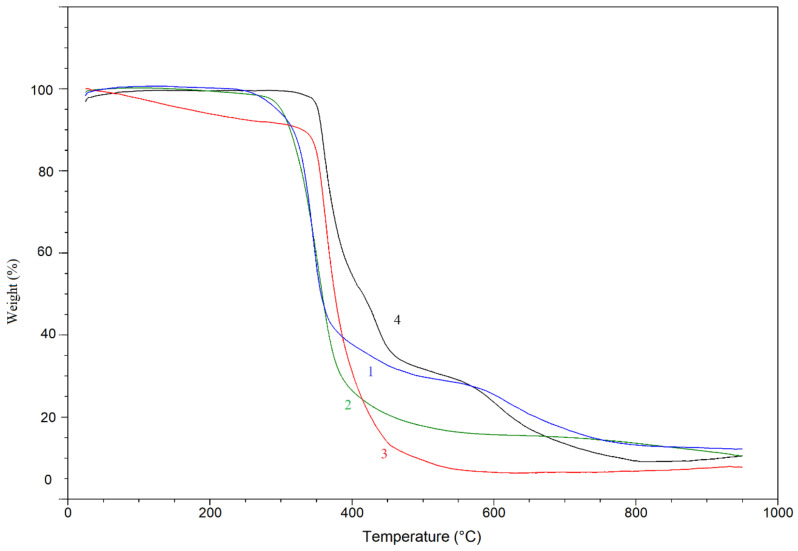
TGA for epoxy samples cured with: HCACTP (curve 1), DTCATP (curve 2), EDA (curve 3) and DPTA (curve 4).

In Figure 13, TG thermograms for the prepared epoxy materials are depicted. The results of analysis are summarized in Table 7. TGA profiles revealed that the DGEBA thermosets cured with HCACTP and DTCATP exhibited a lower thermo-oxidative stability in the comparison with the DPTA-cured epoxy resin. At the same time, the DGEBA thermosets cured with HCACTP and DTCATP were found to be more stable than the EDA-cured sample. The estimated limiting oxygen index (LOI) was calculated for all testing samples from the char yield results at 900 °C, in which weight of the residue practically remains constant. The lower the LOI value reflects the easier ignition of materials. As reported in Table 7, the samples with cyclotriphosphazenes derivatives have LOI numbers always higher than commercially used aliphatic amines. The highest LOI was calculated for sample cured with HCACTP derivative. The LOI number was found to be higher for 6.4% or 4.0% in the comparison with EDA or DTPA, respectively.

The combustion behavior and the flame resistance of the prepared epoxy materials were characterized with the following parameters: time to ignition (TTI), peak heat release rate (pHRR), total heat release (THR), maximum average rate of heat emission (MARHE), total smoke production (TSP) and mass loss rate (MLR). TTI is often used to determine the influence of a flame retardant on the ignitability of a material. As summarized in Table 8, TTI values of the epoxy materials cured with HCACTP and DTCATP, were significantly increased in the comparison with other samples. It has to be noted that the DTCATP-cured epoxy resin achieved two times higher TTI than both amine-cured thermosets. Such behavior suggests a considerable flame resistance effect of both phosphazene-based curing agents in epoxy materials.

**Figure 14:**
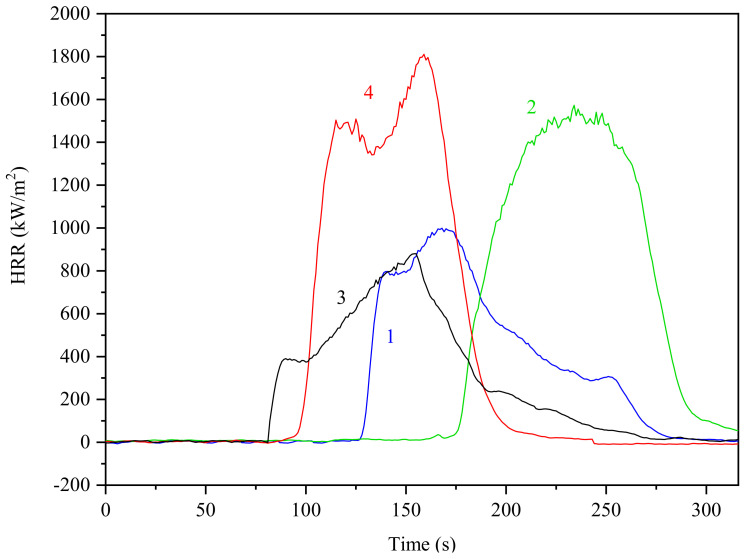
Heat release rate (HRR) curves of epoxy samples cured with: HCACTP (curve 1), DTCATP (curve 3), EDA (curve 2) and DPTA (curve 4).

The heat release rate (HRR) parameter is one of the most important parameter to quantify the size of fire and effective flame resistance of systems normally exhibits a lower HRR values. When comparing the HRR of the tested epoxy materials (see in Figure 14), it is evident that the application of both phosphazene-based derivatives significantly decreased values of HRR in the comparison with samples with amine curing agents. Similarly, MARHE and THR parameters for the epoxy resin cured with cyclotriphosphazene derivatives were found to be lower in the comparison with those of the amine-cured epoxy materials. These results may be attributed to the decomposition of phosphazene, which resulted in the formation of phosphorus-rich char layer imparting an increased heat insulation and thermal stability. The formed char protected barrier probably inhibited heat and oxygen transfer into the interior of the epoxy material and suppressed the transfer of flammable volatiles into flame zone during the combustion process. Thus, the quantity of released heat and the intensity of combustion pyrolysis reactions were decreased.

The results revealed that the DTCATP-cured epoxy resin exhibited the highest TSP value, whereas the HCACTP-cured epoxy resin was shown to produce the lowest quantity of smoke. It can be supposed that the smoke production is partly associated with nitrogen concentration in the cured epoxy material (see Table 9), which is responsible for ammonia release. Moreover, this phenomenon may be contributed to strength, compactness and porosity of the created char layer which affects the transfer of heat and the quantity of released smoke. Hence, a continuous and porous char layer was probably formed during combustion of the DTCATP-cured epoxy resin, while burning of the HCACTP-cured DGEBA resulted in a continuous char shield. Moreover, the results of MLR also confirmed the flame-retarding behavior of both phosphazene-based curing agents. In comparison with the amine-cured epoxy materials, the cyclophosphazene-cured thermosets exhibited a significantly delayed mass loss rate. Based on the results discussed above, it can be concluded that both cyclotriphosphazene derivatives imparted a pronounced flame retarding effect into the DGEBA-based epoxy materials, where the incorporation of HCACTP as a curing agent provided a higher flame retardancy than the curing with DTCATP.

In comparison with the literature [32,56,83,84,85,86], pHRR of bisphenol A bis(diphenyl phosphate) oligomer (phosphazene derivate) [83] was reported to be worse in the comparison with the cyclotriphosphazene-based curing agents. Another work dealt with the usage of a modified epoxy resin with phosphoric acid [84]. pHRR values were detected to be 650 kW/m^2^ and 590 kW/m^2^ (much higher values than for HCACTP and DTCATP as reported in Table 8). The application of carbon nanotubes combined with hexaphenoxycyclotriphosphazene [85] resulted also into the worse pHRR values. The best pHRR result (501 kW/m^2^) was obtained for the system named EP1 (containing 2.0 wt % of P, 14.92 wt %) of hexaphenoxycyclotriphosphazene and 1 wt % of carbon nanotubes. Tested compound containing phosphaphenanthrene and phosphazene groups [56] showed the best pHRR value of 383 kW/m^2^. Phosphazene-based flame retardant with active amine groups of polyphosphazene resulted in the product with pHRR value of 474.78 kW/m^2^ and TSP value of 19.44 m^2^ [32]. pHRR value of 426 kW/m^2^ was reported in the case of usage of monomer with six functional epoxy groups combining by eugenol with hexachlorocyclotriphosphazene [86].

Two novel flame retardants have been successfully prepared. Compared to the traditional curing agents such as DPTA and EDA, DCCATP and HCACTP-cured epoxy materials showed better flammability parameters including TSP. Thus, the usage of DCCATP or HCACTP may help to preserve the environment by decreasing the amount of released harmful pollutants into atmosphere in case of a fire accident. The prepared materials based on the amino-functionalized cyclotriphosphazene derivatives could find application as e.g., protective coatings, high-performance plastics and adhesives.

## 4. Conclusions

In the present work, two novel amino-functionalized cyclotriphosphazene derivatives, namely HCACTP and DTCATP, were successfully synthesized through nucleophilic substitution of hexachlorocyclotriphosphazene. Both derivatives were tested as curing agents for a conventional DGEBA epoxy resins. Epoxy resins with DTCATP was found to provide a more densely crosslinked epoxy material, probably due to the presence of primary amine groups providing a higher reactivity and crosslinking capacity. The results of cone calorimeter measurements revealed that both derivatives significantly improved flame retardancy of the epoxy resin. HCACTP was shown to be a more effective flame retardant in terms of HRR and TSP parameters, while DTCATP provided higher TTI. The pronounced flame-retardant effects of the amino-functionalized cyclotriphosphazene derivatives showed their high potential as curing agents for conventional epoxy resins intended for fire-resistant building and construction applications.

## Figures and Tables

**Table 1:** Dosages and precuring conditions of diglycidyl ether of bisphenol A (DGEBA) and the curing agents.

Curing Agent	*H_ekv_* (g)	DGEBA Amount (g)	Curing Agent Amount (g)	Temperature (°C)	Time (h)
HCACTP	120.65	100	69.9	100	4
DTCATP	69.95	100	40.6	70	4
EDA	15.03	100	8.7	100	4
DPTA	26.20	100	15.2	100	4

**Table 2:** Elemental analysis results of the synthetized cyclotriphosphazene derivatives.

Element	HCACTP		DTCATP	
	Theoretical (wt %)	Experimental (wt %)	Theoretical (wt %)	Experimental (wt %)
C	59.73	59.82	51.51	51.60
H	17.41	17.02	9.37	9.33
N	10.02	10.42	20.95	20.95
P	12.84	12.74	18.17	18.12

**Table 3:** Amine number and purity of the synthetized cyclotriphosphazene derivatives.

Sample	Amine Number (mg KOH/g)		Purity (%)
	Theoretical	Experimental	
HCACTP	465.00	467.30	99.50
DTCATP	601.18	602.50	99.78

**Table 4:** TGA results of HCACTP and DTCATP in the relation to the respective stage of thermo-oxidative degradation.

Stage	Temperature at Rapid Weight Loss (°C)		Weight Loss (wt %)		Corresponding Molecular Weight Loss (g/mol)	
	HCACTP	DTCATP	HCACTP	DTCATP	HCACTP	DTCATP
1st	99.79	104.65	10.3	3.3	74.2	18.2
2nd	343.94	315.42	53.9	56.7	390.4	317.6
3rd	654.33	645.18	35.0	37.2	253.5	208.2

**Table 5:** TGA results of HCACTP and DTCATP thermo-oxidative degradation.

Sample	Temperature at Characteristic Weight Loss (°C)		Chair Yield at 680–950 °C (wt %)
	5 wt %	10 wt %	
HCACTP	99.2	126.9	8.12
DTCATP	247.2	283.2	6.13

**Table 6:** Crosslinking density and glass transition temperature of epoxy materials differing in the used curing agent.

Curing Agent	Crosslinking Density (moles/cm^3^)	*T_g_* (°C)
HCACTP	7.13 × 10^−5^	87.7
DTCATP	8.13 × 10^−5^	101.3
EDA	9.38 × 10^−5^	119.0
DPTA	9.32 × 10^−5^	115.4

**Table 7:** Thermogravimetry analysis (TGA) results of the cured epoxy materials.

Curing Agent	Temperature at the Onset of Degradation (°C)	Temperature at Characteristic Weight Loss (°C)		Char Yield at 600 °C (wt %)	Char Yield at 900 °C for Calculation LOI (wt %)	Estimated LOI (vol. %) *
		5 wt %	10 wt %			
HCACTP	311.5	306.4	321.9	24.8	19.5	25.3
DTCATP	303.8	300.0	312.8	15.6	11.6	22.2
EDA	30.8	63.8	197.1	7.8	3.6	18.9
DPTA	350.5	355.5	359.4	24.7	9.7	21.3

* Calculated using the equation: LOI = 17.5 + 0.4 (CY) [82].

**Table 8:** The results of testing of the cured epoxy resins using cone calorimeter.

Testing Parameter	Curing Agent			
	HCACTP	DTCATP	EDA	DPTA
TTI (s)	120	166	85	80
pHRR (kW/m^2^)	186.5	243.1	731.1	457.2
MARHE (kW/m^2^)	371.2	390.5	436.0	414.3
THR (MJ/m^2^)	79.1	88.7	116.2	102.5
TSP (m^2^)	9.2	14.1	12.6	11.0
MLR (g/s)	0.03	0.04	0.11	0.07
Average specific MLR (g/s·m^2^)	22.7	31.7	59.7	51.5

**Table 9:** Content of individual elements in the cured epoxy materials.

Curing Agent	Element Concentration (wt %)				
	P	N	O	C	H
HCACTP	10.39	14.10	3.58	62.46	9.47
DTCATP	12.73	17.27	4.38	56.77	8.89
EDA	0	12.16	13.89	65.19	8.75
DPTA	0	13.94	10.62	65.75	9.70
